# Molecular Epidemiology of HIV-1 in African Countries: A Comprehensive Overview

**DOI:** 10.3390/pathogens9121072

**Published:** 2020-12-21

**Authors:** Marta Giovanetti, Massimo Ciccozzi, Cristina Parolin, Alessandra Borsetti

**Affiliations:** 1 Reference Laboratory of Flavivirus, Oswaldo Cruz Institute, Fundação Oswaldo Cruz, Rio de Janeiro 21040-900, Brazil; marta.giovanetti@ioc.fiocruz.br; 2 Medical Statistics and Molecular Epidemiology, University Campus Bio-Medico of Rome, 00128 Rome, Italy; m.ciccozzi@unicampus.it; 3 Department of Molecular, Medicine University of Padova, 35121 Padova, Italy; cristina.parolin@unipd.it; 4 National HIV/AIDS Research Center, Istituto Superiore di Sanità, 00162 Rome, Italy

**Keywords:** HIV-1, Africa, subtypes, circulating recombinant form (CRF), recombinant

## Abstract

The human immunodeficiency virus type 1 (HIV-1) originated in non-human primates in West-central Africa and continues to be a major global public health issue, having claimed almost 33 million lives so far. In Africa, it is estimated that more than 20 million people are living with HIV/Acquired Immunodeficiency Syndrome (AIDS) and that more than 730,000 new HIV-1 infections still occur each year, likely due to low access to testing. The high genetic variability of HIV-1, due to a fast replication cycle and high mutation rate, may cause the generation of many viral variants in a single infected patient during a single day. Therefore, the active monitoring and characterization of the HIV-1 subtypes and recombinant forms circulating through African countries poses a significant challenge to more specific diagnoses, treatments, care, and intervention strategies. In this review, a concise characterization of all the subtypes and recombinant forms circulating in Africa is presented to highlight the magnitude of the HIV-1 threat among the African countries and to understand virus genetic diversity and dispersion dynamics better.

## 1. Introduction

The Human Immunodeficiency Virus type 1 (HIV-1) is one of the world’s most serious public health challenges [1,2]. Soon after the HIV-1 epidemic began, the virus dispersed globally, its progress enhanced by stigma discrimination and widespread inequalities that are currently a major barrier to ending Acquired Immunodeficiency Syndrome (AIDS) [1,2,3]. It is rapidly expanding into geographic areas that were relatively spared from the epidemic and strengthening its presence in countries where AIDS is already the leading cause of death [1,2]. At the end of 2020, the Joint United Nations Program on HIV/AIDS (UNAIDS) estimates there are 38 million people living with HIV/AIDS worldwide. Despite new HIV-1 infections having been reduced by 23% since 2010, Sub-Saharan Africa remains the most affected region, accounting for an estimated 69% of all people living with HIV/AIDS, where gender-based violence and inequalities continue to drive the epidemic [1,2,4,5,6]. In African countries, 25.7 million people are infected with HIV-1, which also accounts for almost two-thirds of the global total of new HIV-1 infections. In 2020, about 1.5 million people were newly infected with HIV-1 in Africa [1,2]. The AIDS epidemic varies dramatically from northern to southern African states. Northern Africa has significantly lower prevalence rates, as their populations typically engage in fewer high-risk cultural patterns that promote virus spread [1,2,7,8]. Southern Africa is the worst affected region on the continent, with approximately 25% of new infections. Heterosexual contacts are the main route of transmission in Africa, and sex work and sexual violence contribute significantly to the spread of the disease [1,9]. Africa also bears the highest burden of HIV-1 in terms of genetic diversity. This review aims to summarize the distribution of HIV-1 subtypes and recombinant forms in African countries. 

## 2. HIV Origin

HIV-1 originated in non-human primates in West-central Africa through a process known as zoonosis, transferring to humans in the early 20th century [10,11,12]. Retrospective studies trace the origin to the Democratic Republic of Congo, and from there, HIV-1 spread to other areas in sub-Saharan Africa and West Africa [12,13,14]. The Human Immunodeficiency Virus type 2 (HIV-2) was also introduced through cross-species transmissions of simian immunodeficiency virus from non-human primates to humans [14,15]. HIV-2 is mainly found in West Africa, where an estimated 1-2 million people are infected, although its prevalence is decreasing [16]. HIV-2 is less pathogenic than HIV-1, and the progression to immunodeficiency occurs more slowly when compared to HIV-1 [16]. HIV-2 includes nine distinct subtypes (A-I), group A being the most prevalent, followed by group B and one circulating recombinant form (CRF) CRF01_AB [15,16]. 

## 3. HIV-1 Genetic Diversity

HIV-1 diversified extensively during worldwide dissemination, undergoing constant molecular evolution [17,18,19,20]. Each HIV-1 replication cycle generates approximately one mutation per genome. This extraordinary virus mutation rate is largely ascribed to four factors: i) the activity of reverse transcriptase, which favors the accumulation of transcription errors that the enzyme is unable to correct since it lacks 3′ to 5’ exonuclease activity; ii) the high rate of viral replication; iii) the propensity of HIV-1 to undergo genetic recombination during replication; iv) host selective immune pressures [19,20,21,22,23]. As a consequence, wide genetic divergence within the viral population may arise quickly, generating closely related but distinct viral variants. This variability not only involves the viral population infecting different individuals, known as interhost variability (2–5%) but also that present within the same individual, the intrahost variability (6–19%) [23,24,25]. The variation between subtypes ranges between 20 and 35%, depending on the subtypes and genetic regions examined. Inter-subtype sequence diversity is in the order of 8–17% but can be as high as 30% [23,24,25]. Infection by different HIV-1 strains, concomitant (co-infection) or sequential (super-infection), may result in recombinant viruses, thus increasing HIV-1 genetic diversity further [26,27,28].

## 4. HIV-1 Subtypes and Recombinants

**Figure 1:**
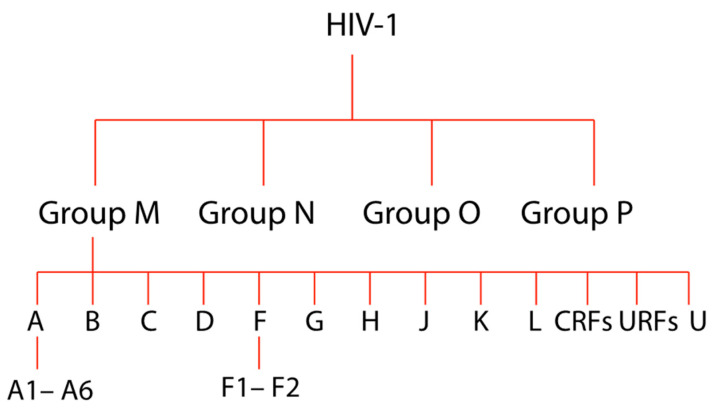
Schematic representation of the evolutionary relationships of human immunodeficiency virus type 1 (HIV-1) groups, subtypes, sub-subtypes, and recombinant forms. “U” for untyped. Worldwide proportions of HIV-1: subtype C (46.6%), B (12.1%), A (10.3%), G (4.6%), D (2.7%), F, H, J, K (together 0.9%), circulating Recombinant Forms (CRFs) 16.7%, unique Recombinant Forms (URFs) 6.1%. N, O, and P groups are extremely rare.

Using complete viral genome sequencing but based mainly on the characterization of the env and/or gag genomic regions, it is possible to distinguish four phylogenetic HIV-1 groups: M (major), O (outlier), N (non-M/non-O), and the most recent group P [29,30]. Unlike group M viruses, which have dominated the global HIV-1 pandemic since its inception, the other groups N, O, and P have not been disseminated widely and only comprise ~1–2% of all HIV-1 infections [29,30,31]. Group M includes more than 95% of all isolated strains and can be classified further into nine subtypes (A–D, F–H, J, K), six A (A1–A6), and two F (F1–F2) sub-subtypes areas (Figure 1) [29,30,31,32,33].

In the Democratic Republic of Congo, three divergent HIV-1- strains have been isolated to form a new distinct subtype (subtype L) [34,35]. The intra-subtype genetic variation ranges from 8–20%, while variation between subtypes is usually 17–35%, depending on the subtypes and genome regions examined [19,24,26]. By phylogenetic analysis, discrete and randomly distributed breakpoints between genomic regions can be identified in recombinant viruses [19,24,26]. The process of genetic recombination is the basis for the emergence of the so-called “mosaic” strains designated as circulating recombinant forms (CRF) [19,24,26,29] and defined as full-length or near full-length HIV-1 sequences that are found in at least three epidemiologically unlinked individuals [24,29]. CRFs are quite common; as of November 2020, 106 CRFs have been registered by the Los Alamos National Laboratory [36,37,38]. The CRFs are named in the order in which they are reported and by the recombinant subtypes forming the genomic structure. The most prevalent CRFs in the global HIV-1 epidemic are CRF01_AE and CRF02_AG. The first CRF to be identified was CRF01_AE, which is a recombinant of a subtype A and a putative extinct subtype E ancestor, containing subtype A *gag, pol,* and subtype E *vif, vpr, env, nef,* and long terminal repeat (LTR). CRF02_AG is a recombinant of subtypes A and G, bearing subtype A *gag, env*, subtype A/G *pol, tat, rev, nef,* and subtype G LTR [36,37,38]. CRFs that consist of three or more different HIV-1- strains are indicated as cpx for “complex” recombination of several subtypes [24,29]. Unique recombinant forms (URFs) represent a heterogeneous group, consisting of a mixture of subtypes that show unique recombination breakpoints. Unlike the CRFs, they were sampled only once from a single multiply infected individual [19,24,29,39]. A high proportion of URFs has already been described from the Republic of the Congo and West and Central African countries [40]. The AD and AC inter-subtype recombinants have been found in Eastern Africa, where subtypes A, C, and D are the most prevalent [30]. Not all HIV-1 lineages have been well-characterized, and in some cases, the correct classification of virus genome sequences falls outside the known diversity. Thus, these sequences are listed as “U” for untyped [19,26]. Classification of HIV-1 strains remains a complex issue since new viral sequences are continually identified, and the definitions of different HIV-1 subtypes are subject to change [41]. The latest estimate of all HIV-1 infections worldwide in 2010-15 indicates that subtype C is responsible for 46.6% of infections, subtype B for 12.1%, subtype A for 10.3%, subtype G for 4.6%, subtype D for 2.7%, all together F, H, J, K for 0.9%, CRF02_AG for 7.7%, CRF01_AE for 5.3%, other CRFs for 3.7% and URFs for 6.1% [29,30,31,32,33,41]. Globally, subtype B is the most prevalent in the Americas, Europe, and Oceania, subtype C in Southern Africa and India, subtype A in the Soviet Union and parts of East Africa, CRF01_AE in Asia, and CRF02_AG in Western and Central Africa [42,43]. The genetic variability of HIV-1 is able to influence the mode of transmission and interaction between the virus and target cell, the clinical course of the infection itself, and the response to treatment, as indicated by subtype-specific differences in transmission rates and disease progression [42,43,44,45,46,47]. Globally, subtype C is the most prevalent; however, it is unclear whether phenotypic differences exist compared to the other subtypes that permit its enhanced transmission efficiency or pathogenicity [44,45]. Subtype D and CRF01_AE have been associated with faster disease progression. Analysis of a large cohort in Uganda demonstrated a strong difference in survival rates of D vs. A subtype [48]. A large study in China revealed that subjects carrying the CRF01_AE subtype exhibited faster loss of CD4+T cells and HIV/AIDS progression compared with those carrying a non-CRF01_AE subtype [49]. Several factors, including host genetic and immunologic responses, may influence disease progression rate differences between subtypes, and the current evidence for survival is inadequate to draw definitive conclusions. The geographic distribution and dispersal of HIV-1 subtypes and recombinant forms in African regions is a complex and dynamic process involving human migrations, conflict, mobility, geographical isolation, and cultural and sexual factors [49,50,51,52,53,54]. The increasing HIV-1 genetic diversity has significant implications for viral pathogenicity, drug resistance testing, and vaccine development that require up-to-date knowledge regarding the geographic distribution of the HIV-1 subtypes and recombinant forms.

## 5. Distribution of Subtypes and Recombinants in African Regions

**Figure 2:**
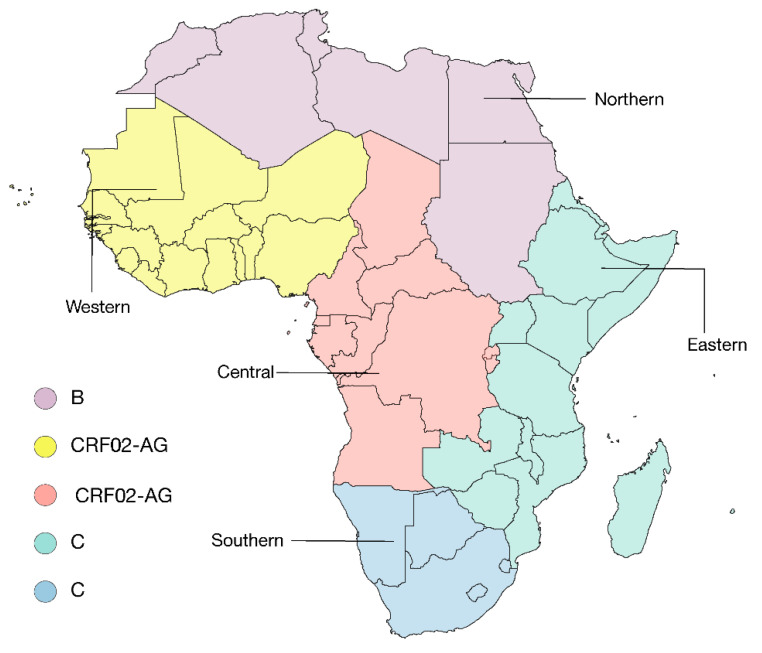
Pattern of the currently dominant genotypes in Africa, 1990–2020. Map of Africa showing the predominant subtypes and/or recombinant forms in the five geographic regions: Northern (Algeria, Egypt, Libya, Morocco, Sudan, *South Sudan, Tunisia), Western (Benin, Burkina Faso, Cabo Verde, Côte d’Ivoire, Gambia, Ghana, Guinea, Guinea-Bissau, Liberia, Mali, Mauritania, Niger, Nigeria, Senegal, Sierra Leone, Togo) Central (Angola, Cameroon, Central African Republic, Chad, the Democratic Republic of the Congo, Equatorial Guinea, Gabon, Burundi, Rwanda), Eastern (Djibouti, Eritrea, Ethiopia, Kenya, Madagascar, Malawi, Mauritius, Mayotte, Mozambique, Réunion, Seychelles, Somalia, Uganda, United Republic of Tanzania, Zambia, Zimbabwe), Southern (Botswana, Saint Helena, eSwatini, Lesotho, Namibia, South Africa). African macro regions are colored according to the dominant genotype. (*) South Sudan was included in Northern Africa.

The HIV-1 distribution on the African continent over time was determined by analyzing a total of 204,573 HIV-1 samples, each with a minimum length of 500 bp, collected over 1990–2020, and available in a public database (Los Alamos: https://www.hiv.lanl.gov/content/index). African countries were aggregated into five macro regions: Northern, Western, Eastern, Central, and Southern, according to the UNAIDS classification, with some modifications taking into account the geopolitical characteristics of the territories and the social, cultural, and religious features. Northern countries have similar cultural norms, while Central countries have a history of armed conflict, with a high number of refugee-related challenges, as well as food insecurity [55]. Western and Eastern countries are grouped based on their geographical location. Countries included in the Southern region have the highest HIV-1 prevalence globally, with approximately 20% of adults living with HIV-1. The respective countries for each region are listed in the legend of Figure 2.

Cumulative analysis of the distribution of HIV-1 (204,573 samples) in Africa, spanning 1990–2020, hence covering the whole period of the pandemic, shows that in Northern Africa, subtype B has predominated (53.04%). In Western and Central Africa, the CRF02_AG has been most prevalent (53.66% and 26.61%, respectively), and in Eastern and Southern Africa, collectively subtype C has predominated (44.15 and 98.44%, respectively). 

**Figure 3:**
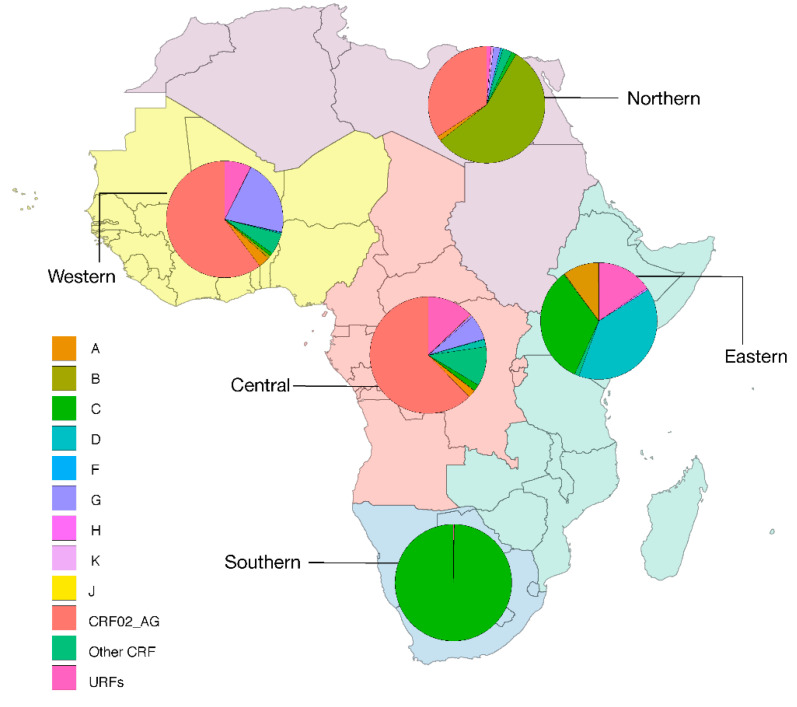
The geographic distribution of HIV-1 subtypes, CRFs, and URFs, 2015–2020. The colors representing the different HIV-1 subtypes and recombinants are indicated in the legend.

Estimation of the macro regional distribution of HIV-1 subtypes, CRFs, and URFs over the most recent five-year period, 2015–2020, is shown in Figure 3. 

Marked differences can be observed between the two maps. The majority of HIV-1 circulating in Northern Africa is subtype B, with a notable increase of CRF02_AG. In Western and Central Africa, CRF02_AG and subtype G predominate, although all major subtypes and many CRFs and URFs are also represented. Eastern Africa is mainly affected by subtypes D, C, A, and by a high proportion of URFs. The epidemic in Southern Africa is almost exclusively due to subtype C. 

**Figure 4:**
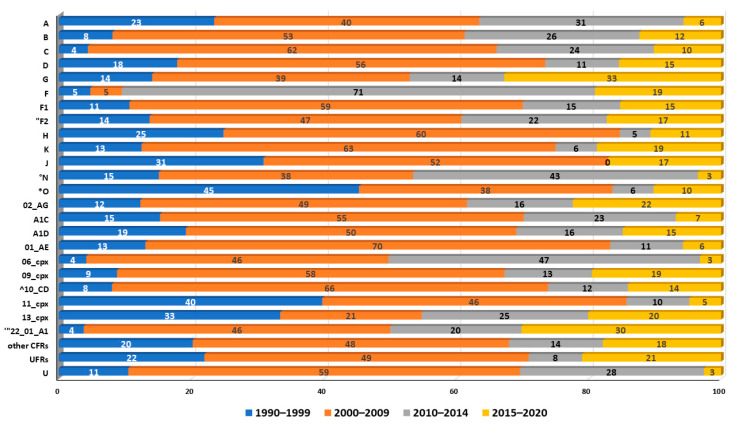
Percentage of the cumulative occurrence for each subtype over the 1990–2020 period. HIV-1 subtypes, CRFs, and URFs in 1990–1999, 2000–2009, 2010–2014, 2015–2020. Subtypes O, F2, N, and CFRs 22_01_A1 and 10_CD are present in the Los Alamos HIV-1 database from 1992, 1993, 1995, 1996, and 1997, respectively. (*) from 1992, (“) from 1993, (°) from 1995, (’’’) from 1996, (^) from 1997.

To examine the changes in the number of all HIV-1 subtypes, CRFs, an UFRs in African regions over 1990–2020 and most recently in the last five years, the dataset was split into four time periods 1990–1999, 2000–2009, 2010–2014, and 2015–2020 (Figure 4).

Overall, the percentage of the cumulative occurrence of subtypes, recombinants, and untyped forms increased between 2000–2009, with the exception of subtype F, group N, and CRF06_cpx and CRF22_01_A1, which expanded between 2010–2020, and of group O that made a substantial contribution between 1990–1999. The last five years, 2015-2020, are characterized mainly by an increase in subtypes G, J, and recombinants CRF02_AG, CRF09_cpx, CRF22_01_A1, URFs.

**Figure 5:**
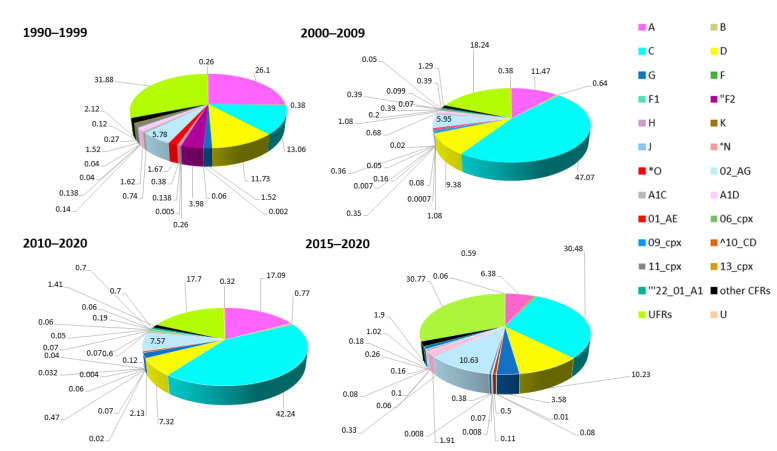
Distribution of HIV-1 subtypes, CRFs, and URFs in 1990–1999, 2000–2009, 2010–2020. Proportion of each subtype relative to the others at the continental level is represented. The colors representing the different HIV-1 subtypes and recombinants are indicated in the legend on the right-hand side of the figure. (*) from 1992, (“) from 1993, (°) from 1995, (’’’) from 1996, (^) from 1997.

The evolution of the contribution of each subtype and recombinant form to the epidemiology in Africa over thirty years is shown in Figure 5.

Specifically, the contribution of each subtype and recombinant form relative to the others over time was analyzed by splitting the dataset into four time periods 1990–1999, 2000–2009, 2010–2020, and 2015–2020. Since the beginning of the epidemic, the majority of HIV-1 circulating in Africa is subtype C, accounting for nearly half (42.4%) of all infections in 2000–2009, followed by proportional fluctuations, in a descending order, of subtypes A, D, G, and also of CRF02_AG. URFs make a substantial contribution throughout all periods Considering the last five years, an increased contribution of recombinants, CRF02_AG (10.63) and URFs (30), as well as a decrease of subtype C (30.48) is observed.

## 6. Summary

Even with a long history of HIV-1 circulation, African countries continue to struggle with effective HIV-1 control and public health response. One major challenge is the active monitoring and characterization of HIV-1 genetic diversity throughout the African continent to enable more specific diagnosis, treatment, care, and intervention strategies. Analysis of HIV-1 distribution in Africa spanning thirty years of the epidemic reveals distinct geographical regions with different distributions of HIV-1 subtypes and recombinant forms. The Northern region is primarily affected by subtype B, the Southern region by subtype C, the Western and Central regions by CFR02_AG and, the Eastern region is dominated by subtype C. Detailed examination of HIV-1 subtype and recombinant distribution, in the most recent period (2015–2020), indicates high subtype diversity and dynamic changes, in some regions. For example, in Northern Africa, subtype B originally predominated, but during the last five years, some recombinant forms, including CRF02_AG and other CRFs, have greatly increased, suggesting an expansion of CRFs in this epidemic. In Southern Africa, subtype C has been most prevalent since 1990. However, a closer analysis of this period showed subtype C slightly decreasing in 2010–2020, despite a substantial decrease in the last 5 years. In this region, home to millions of HIV-1 infected people, many factors may have contributed to the decreasing spread and diversification of subtype C. For example, the rapid scale-up of antiretroviral therapy has reduced HIV-1-related mortality between 2010 and 2017 by 30% (UNAIDS, 2020). The greatest HIV-1 genetic diversity has been found in Western and Central Africa where, as indicated by the relative maps, CRF02_AG is the dominant subtype and co-circulates, in a different proportion, with all of the other subtypes. Moreover, in Eastern Africa, an increase of subtype D and recombinant forms has been observed in the last five years. It is noteworthy that, despite a consistent global CRFs and URFs expansion, less novel recombinants have emerged from Africa, particularly in Central Africa. The proportion of CRFs and URFs over 2010–2020 appears to be quite stable, with only CRF02_AG, CRF09_cpx, A1D, and URFs increasing in 2015–2020. These three recombinants are playing a greater role in the AIDS epidemic in the macro regions where multiple subtypes co-circulate. The significant increase in the number of CRFs and the changing patterns of the geographic distribution of HIV-1 subtypes and recombinant forms over time reveals the necessity of molecular surveillance. The practical implication of HIV-1 variability is its impact on the efficacy of new drugs and treatment strategies. The number of resistant strains that are transmitted may increase, especially in resource limited countries. Considering that drug resistance and susceptibility are influenced by HIV-1 genotypes [56], surveillance is required, particularly in the subtypes, CRFs and URFs that are less common to ensure accurate diagnosis, treatment regimens, and future vaccine development. It is essential to expand analysis of the HIV-1 diversity at the full-length genome level from different countries, taking into consideration epidemiological factors.

## 7. Conclusions

Mutation and recombination are the major forces driving HIV-1 genomic diversity. The continual emergence of new recombinant forms presents an enormous surveillance challenge. Monitoring HIV-1 diversity and dispersal in Africa is critical to understand the impact these strains may have on AIDS diagnosis, treatment, and intervention strategies.
